# Supplementary material for: The impact of grandparent-grandchild interactions on the imagination of aging among Chinese youth groups: The chain mediating role of intergenerational relations and filial piety concept
Source: PLoS One. 2026 Apr 15;21(4):e0345803. doi: 10.1371/journal.pone.0345803 (PMC13082605; doi:10.1371/journal.pone.0345803)
Supplement: S1 Table — (DOCX) [file pone.0345803.s001.docx]

**S1 Table The chain mediation of interpersonal relations and filial piety among grandchildren and youths(Model 1 without covariates)**

|  |  |  |  | **95%CI** | |
| --- | --- | --- | --- | --- | --- |
| **Model** | **Intermediary paths for variables** | **Standardized effect size（β）** | **BootSE** | **BootLLCI** | **BootULCI** |
| Model 1 | D.G.G.I→Y.P.I.A(Total Effect) | 0.2871 | 0.0344 | 0.2196 | 0.3546 |
|  | D.G.G.I→Y.P.I.A(Total Direct effect) | 0.1467 | 0.0403 | 0.0677 | 0.2257 |
|  | Total Indirect Effect | 0.1404 | 0.0311 | 0.0825 | 0.2029 |
|  | D.G.G.I →I.R →Y.P.I.A | 0.0867 | 0.0261 | 0.0378 | 0.1386 |
|  | D.G.G.I→F.P→Y.P.I.A | 0.0343 | 0.0123 | 0.0132 | 0.0606 |
|  | D.G.G.I→I.R→F.P→Y.P.I.A | 0.0194 | 0.0070 | 0.0078 | 0.0350 |

Note. D.G.G.I,Daily grandparent-grandchild interactions; Y.P.I.A,Young people's imagination of aging; I.R,Intergenerational relations; F.P,filial piety
