# Supplementary material for: The impact of grandparent-grandchild interactions on the imagination of aging among Chinese youth groups: The chain mediating role of intergenerational relations and filial piety concept
Source: PLoS One. 2026 Apr 15;21(4):e0345803. doi: 10.1371/journal.pone.0345803 (PMC13082605; doi:10.1371/journal.pone.0345803)
Supplement: S2 Table — (DOCX) [file pone.0345803.s002.docx]

**S2 Table The chain mediation of interpersonal relations and filial piety among grandchildren and youths（Model 2 without covariates）**

|  |  |  |  | **95%CI** | |
| --- | --- | --- | --- | --- | --- |
| **Model** | **Intermediary paths for variables** | **Standardized effect size（β）** | **BootSE** | **BootLLCI** | **BootULCI** |
| Model 2 | R.G.G.I→Y.P.I.A（Total Effect） | 0.2482 | 0.0348 | 0.1799 | 0.3165 |
|  | R.G.G.I→Y.P.I.A(Total Direct effect) | 0.1295 | 0.0376 | 0.0557 | 0.2033 |
|  | Total Indirect Effect | 0.1187 | 0.0248 | 0.0717 | 0.1688 |
|  | R.G.G.I→I.R→Y.P.I.A | 0.0798 | 0.0212 | 0.0392 | 0.1233 |
|  | R.G.G.I→F.P→Y.P.I.A | 0.0152 | 0.0083 | 0.0011 | 0.0333 |
|  | R.G.G.I→I.R→F.P→Y.P.I.A | 0.0237 | 0.0073 | 0.0110 | 0.0397 |

Note. R.G.G.I,Ritualistic grandparent-grandchild interactions;Y.P.I.A,Young people's imagination of aging;I.R,Intergenerational relations; F.P,filial piety
