# Supplementary material for: The impact of grandparent-grandchild interactions on the imagination of aging among Chinese youth groups: The chain mediating role of intergenerational relations and filial piety concept
Source: PLoS One. 2026 Apr 15;21(4):e0345803. doi: 10.1371/journal.pone.0345803 (PMC13082605; doi:10.1371/journal.pone.0345803)
Supplement: Attachment — Submitted filename: Response to Reviewers.docx [file pone.0345803.s004.docx]

Dear editor and Reviewers,

Thank you very much for the opportunity to revise our manuscript entitled “The Impact of Grandparent–Grandchild interactions on the Imagination of Aging among Chinese Youth Groups: The Chain Mediating Role of Intergenerational Relations and Filial Piety Concept” (Manuscript No. PONE-D-25-46690). We sincerely appreciate the time and effort you and the reviewers have devoted to evaluating our work. We have carefully considered all comments from editor/Reviewer #1 and Reviewer #2, and have undertaken substantial revisions to strengthen the manuscript conceptually, methodologically, and substantively.Below, we provide a detailed response to each of their comments. All revisions have been highlighted in blue/red/purple in the revised manuscript.

**—Response to editor**

***Comment 1. Please ensure that your manuscript meets PLOS ONE's style requirements, including those for file naming. The PLOS ONE style templates can be found at https://journals.plos.org/plosone/s/file?id=wjVg/PLOSOne_formatting_sample_main_body.pdf and <https://journals.plos.org/plosone/s/file?id=ba62/PLOSOne_formatting_sample_title_authors_affiliations.pdf>***

***Response:***

We have revised the format in accordance with the template requirements of PLOS ONE.

***Comment2. Thank you for stating the following financial disclosure: “This research was funded by Guangxi Philosophy and Social Science Foundation Project (Nos:23FSH028)”Please state what role the funders took in the study.  If the funders had no role, please state: "The funders had no role in study design, data collection and analysis, decision to publish, or preparation of the manuscript." If this statement is not correct you must amend it as needed. Please include this amended Role of Funder statement in your cover letter; we will change the online submission form on your behalf.***

***Response：***

This research was funded by the Guangxi Philosophy and Social Science Foundation (No. 23FSH028) and the Guangxi Normal University Project (No. 2024PY004). The corresponding author of this paper is the principal investigator of the project, and the first author was the primary contributor to its implementation. The survey was conducted with support from these projects, and the article processing charges (APCs) are also covered by them. Thus, the study received full financial support from both projects.

We have already provided a detailed explanation in the cover letter.

***Comment3. Please note that your Data Availability Statement is currently missing the repository name. If your manuscript is accepted for publication, you will be asked to provide these details on a very short timeline. We therefore suggest that you provide this information now, though we will not hold up the peer review process if you are unable.***

***Response：***

We have now provided the name and link to the database.The data that support the findings of this study are available on request from OPEN ICPSR(https://www.openicpsr.org/openicpsr/workspace?path=openICPSR(Project ID:openicpsr-222102)).

***Comment4. Please ensure that you refer to Figures 1 to 4 in your text as, if accepted, production will need this reference to link the reader to the figure.***

***Response：***

We have marked the figures and tables at the relevant positions in the text and cross-checked by two authors

***Comment5. We note you have included a table to which you do not refer in the text of your manuscript. Please ensure that you refer to Tables 1 and 2 in your text; if accepted, production will need this reference to link the reader to the Table.***

***Response*：**

We have labeled Table 1 and Table 2 at the relevant points in the text.

***Comment6. Please include captions for your Supporting Information files at the end of your manuscript, and update any in-text citations to match accordingly. Please see our Supporting Information guidelines for more information: http://journals.plos.org/plosone/s/supporting-information.***

***Response：***

We have added a title for supporting information at the end of the article，Please refer to the relevant sections of the article for specific details.

***Comment7. If the reviewer comments include a recommendation to cite specific previously published works, please review and evaluate these publications to determine whether they are relevant and should be cited. There is no requirement to cite these works unless the editor has indicated otherwise.***

***Response：***

We have cited the literature based on the actual needs of the article, and there are no redundant or irrelevant citations.

**—Below are the responses to Reviewer 1 and Reviewer 2, with the corresponding revisions in the text highlighted in blue or red.**

***Response to Reviewer 1***

***Comment 1:The abstract effectively summarizes the study, but it can be enhanced by simplifying some of the language and focusing on the core findings more directly. Additionally, providing a bit more context on the cultural implications of filial piety could help readers unfamiliar with the topic understand the broader significance.***

***Response：***

We fully agree that simplifying language, centering core findings, and adding cultural context for filial piety would enhance clarity and accessibility for all readers—particularly those unfamiliar with Chinese cultural norms.

We have revised the abstract to address the key suggestions: simplifying language, centering core findings more directly, and enhancing cultural context for filial piety to improve accessibility for readers unfamiliar with Chinese cultural norms.

The revised abstract now foregrounds the cultural framework of Confucian filial piety, explicitly defining it as a core norm centered on respecting elders and intergenerational responsibility, while tightly linking it to China’s population aging context.

We have refined the academic expressions, elaborated on the effects of daily communication and ritualized interaction—the core finding of the study—and clarified the pivotal roles of intergenerational relations and filial piety in both the dual mediating effect and the serial mediating mechanism. The revised version maintains brevity while emphasizing practical implications, ensuring readers grasp the study’s core contributions at a glance.

The following is the final revised abstract, which has been incorporated into the manuscript and appears **on【Lines 18 to 29 of the text】.**

“Centered on respecting elders and intergenerational responsibility, Confucian filial piety culture serves as a core cultural norm in Chinese society. This study integrates this cultural framework with the context of China’s rapid population aging to explore how grandparent-grandchild interactions within Chinese families shape young adults’ aging imagination. Based on a survey of 778 Chinese youth aged 18-35, the findings show that both daily and ritualistic interactions positively predict young adults’ aging imagination, and daily interactions has a stronger predictive effect. Intergenerational relations and filial piety not only serve as independent mediators but also constitute a chain mediating pathway, indicating that emotional bonds and cultural values synergistically foster young people’s positive aging imagination. This study suggests that efforts to strengthen intergenerational interactions and integrate culturally rooted aging education are critical for promoting positive aging attitudes among youth.”

***Comment 2. The research questions and hypotheses are central to the structure of this study, but there seems to be a mismatch between them that could confuse readers or undermine the clarity of the study's design. Specifically:***

***Research Question 1 (Q1) focuses on routine (daily) grandparent-grandchild communication and its influence on young people's perceptions of aging.***

***Research Question 2 (Q2) looks at ritualistic communication and its impact on the same outcome.***

***However, the hypotheses (H1 and H2) do not fully align with these research questions. Both hypotheses center on intergenerational relations as a mediator, but they do not clearly differentiate between how routine and ritualistic communication might work differently through these relations.***

***Response：***

We fully agree your opinion. To address this problem, we have revised the study’s conceptual framework to unify all research aims under hypotheses, ensuring clarity, consistency, and rigor in the study design.

Specific Revisions:

Eliminated standalone research questions (Q1-Q2): We replaced the question-based framing with explicit hypotheses to directly link each communication type (daily/ritualistic) to the outcome (aging imagination) and clarify their unique pathways.

Restructured and renumbered hypotheses for alignment:

Original Q1 (daily interactions→aging imagination) is now Hypothesis 1: Daily interactionss between grandparents and grandchildren significantly influences the aging imagination of young people. **【See line 173 of the text】.**

Original Q2 (ritualistic interactionss→aging imagination) is now Hypothesis 2:Ritualistic interactions between grandparents and grandchildren significantly influences the aging imagination of young people. **【See line 175 of the text】.**

Original H1-H6 are renumbered as Hypotheses 3-8, with each explicitly specifying the communication type they address to eliminate ambiguity:

H3:Intergenerational relations mediate the association between daily grandparent-grandchild interactions and young peoples’aging imagination of aging. **【See line 205 of the text】.**

H4: Intergenerational relations mediate the association between ritualistic grandparent-grandchild interactions and young adults’ aging imagination.**【See line 207 of the text】.**

H5: Filial piety mediates the association between daily grandparent-grandchild interactions and young adults’ aging imagination.**【See line 246 of the text】.**

H6: Filial piety mediates the association between ritualistic grandparent-grandchild interactions and young adults’ aging imagination.**【See line 248 of the text】.**

H7: Intergenerational relations and filial piety sequentially mediate the association between daily grandparent-grandchild interactions and young adults’ aging imagination.**【See line 285 of the text】.**

H8: Intergenerational relations and filial piety sequentially mediate the association between ritualistic grandparent-grandchild interactions and young adults’ aging imagination.**【See line 288 of the text】.**

***Comment 3. The discussion effectively summarizes the study's findings and their contributions to the field, but it would benefit from a more critical and in-depth analysis of the results. Specifically, the discussion could explore the following areas more deeply:***

***3.1 Theoretical Implications: While the study connects its findings with existing theories like stereotype internalization theory, it would be valuable to expand on how the findings either challenge or extend these theories. For example, how do the specific mechanisms (daily vs. ritualistic communication) align with or contradict existing models of intergenerational communication and aging? Are there any contradictions or nuances that haven't been addressed yet?***

***3.2 Cultural Context: The study is grounded in the Chinese family context, which is a significant contribution. However, a more critical exploration of filial piety in relation to aging imagination would enrich the discussion. Given that filial piety is a cultural value that varies significantly across different societies, how might these findings differ in other cultural contexts? Could there be alternative interpretations or dimensions of filial piety that the study has not fully addressed?***

***Response:***

Thank you for your profound insights on the discussion section of the manuscript. We fully agree that these comments will significantly enhance the quality of the paper. Below is a detailed explanation of the targeted revisions we have made in response to these points.

We engaged in theoretical dialogue with Stereotype Embodiment Theory (SET) by analyzing how our findings validate, extend, and revise the theory:

(1) Validating the Core Proposition of Stereotype Embodiment Theory (SET)

We confirmed the core tenet of SET regarding the early internalization of age-related beliefs (Levy, 2009). Our findings reveal that grandparent-grandchild communication, as an early source of age-related experiences, influences young adults’ aging imagination—an explicit manifestation of internalized age schemas (Diel & Wahl, 2024). This provides empirical support for SET’s claim that "age stereotypes become embodied through life course processes."

(2) Extension: From Passive Internalization to Active Relational Construction

SET primarily conceptualizes stereotype internalization as a unidirectional process driven by macro-social narratives (e.g., media portrayals; Wu, 2021). Our results challenge this perspective by demonstrating that grandparent-grandchild communication operates through the mediating role of intergenerational relationship quality. High-quality emotional bonds encourage young adults to proactively engage with and empathize with their grandparents, transforming abstract social stereotypes into concrete, positive aging imaginations (e.g., the transmission of wisdom). This finding extends SET by illustrating, at the family level, how positive intergenerational interactions and relationships can mitigate negative aging perceptions.

(3) Cultural Revision of Stereotype Embodiment Theory (SET)

This study also revises the cultural applicability of SET by contextualizing it within Chinese filial piety culture. SET’s theoretical framework and empirical validation are rooted in Western societies, with limited elaboration on specific Eastern cultural contexts. Our research demonstrates that the process of stereotype internalization is intertwined with cultural norms. In individualistic Western cultures, aging imagination may be more heavily influenced by social welfare systems and media discourse. In the Chinese context, familism serves as the foundation for understanding Chinese society (Yu, 2023), and filial piety (xiao) is not merely an external behavioral norm but a deeply ingrained cultural cognitive schema encompassing intergenerational responsibility, respect for elders, and the meaning of life (Yeh & Bedford, 2003). Our findings show that, through the mediating role of filial piety, young adults internalize positive perceptions of aging during intergenerational interactions, offering a culture-specific perspective to SET.Furthermore, the chain mediating effect of intergenerational relations and filial piety confirms that close emotional bonds lay the groundwork for the voluntary, affect-driven internalization of filial norms. In turn, internalized filial values guide and elevate intergenerational interactions, strengthening emotional connections. This finding aligns with Family Systems Theory’s emphasis on emotional interdependence and Intergenerational Solidarity Theory’s focus on normative integration, indicating that the formation of positive age beliefs is the result of the synergistic interplay between affectual solidarity and normative solidarity in micro-level family practices.

In summary, this study validates and extends Stereotype Embodiment Theory by shifting the research focus from macro-society to micro-family contexts, from passive reception to active construction, and from universal psychological mechanisms to culture-specific pathways. It offers a novel framework for understanding the developmental mechanisms of age cognition.

**【The specific content can be found on Lines 701 to 767 of the text】**

“This study engages in theoretical dialogue with Levy’s Stereotype Embodiment Theory (SET) through an analysis of the formation mechanism of aging imagination among young people.......In summary, this study validates and extends Stereotype Embodiment Theory (SET). It shifts the research focus from macro-level societal contexts to micro-level family dynamics, from passive reception to active construction, and from universal psychological mechanisms to culture-specific pathways. Consequently, this paper offers a novel framework for understanding the developmental mechanisms underlying age-related cognition.”

***Comment 4. The study discusses a phenomenon in Chinese society, which is valuable. However, it would be helpful to more explicitly explain why this specific focus on Chinese society is important and how the findings contribute to the existing body of literature. For example, what unique insights can this study offer in the context of Chinese cultural values, and how does it extend or challenge existing research on intergenerational communication and aging perceptions? A clearer articulation of the study's contribution will strengthen the significance of the findings and their relevance to broader discussions in the field.***

***Response:***

We clarify the significant theoretical and practical implications of this study within the Chinese context:

First of all, Theoretically, we lay the foundational groundwork for the novel concept of "aging imagination." Through a review of Chinese literature, we identified that while the term "aging imagination" has been previously mentioned by some researchers, it lacks concrete and systematic elaboration. To address this gap, we propose a new definition of the concept by integrating insights from the Social Psychological Aging (SPA) theory in Western scholarship. We conceptualize "aging imagination" as an individual’s psychological representation and narrative projection of their future old age, encompassing anticipated physical changes, social roles, life trajectories, and the meaning of late-life existence. Distinct from the Social Psychological Aging (SPA) theory, which primarily explores how individuals evaluate their current aging process, aging imagination focuses on how individuals envision their own aging journey. As a preparatory self-relevant schema, aging imagination bridges the social representations of aging, self-perceptions of aging, and aging-related behaviors.By anchoring the concept in SPA theory, which emphasizes the interplay between individual cognitions, social contexts, and aging processes, our definition bridges the theoretical gap between Chinese and Western aging research, providing a unified conceptual framework for future studies on youth’s aging-related perceptions.**【The specific content is on Lines 55 to 77 of the text】**

**“In the Chinese context, researchers have proposed the concept of “aging imagination”, yet its specific connotations remain undelineated. It has only been described as the perceptions of old age that non-elderly individuals gradually form through interactions with older adults......Aging imagination serves as a preparatory self-cognitive framework that connects societal dominant perceptions of aging, individuals’ personal views on aging, and behaviors when confronting aging.”**

Secondly, Rooted in China’s familistic culture, grounded in filial piety and intergenerational cohabitation, this study establishes a unique context where aging is deeply intertwined with family dynamics (Yue & Zhang, 2018). Unlike Western research that often frames aging as an individual or societal issue, this study emphasizes the family as a pivotal site for young adults’ “aging socialization.” It is within this familial sphere that aging education can be effectively facilitated through the emotional closeness between grandparents and grandchildren, as well as the internalization of filial piety values.

**【The specific content is on Lines 742 to 762 of the text】**

“In individualism-oriented Western cultures, aging imagination may be more strongly shaped by social welfare systems [73] and media representations [74]. In contrast, within China’s collectivism-rooted cultural context, familialism serves as the foundational framework for understanding Chinese society[75]. Filial piety in Chinese culture is not merely an external behavioral norm but a deeply ingrained cultural cognitive schema encompassing intergenerational responsibilities, the value of respecting elders, and the meaning of life[46].This study finds that young adults internalize positive perceptions of aging through intergenerational interactions, with filial piety beliefs functioning as a mediating mechanism. This finding directly challenges the negative narratives prevalent in Western-centric research that equate “aging” with “frailty” and “burden”[16]. By offering a culture-specific perspective on SET, it suggests that the theory may manifest in distinct forms and operate through different mechanisms across cultural contexts. Furthermore, the serial mediating effect of intergenerational relationships and filial piety beliefs indicates that close intergenerational bonds lay the groundwork for the voluntary, emotional internalization of filial norms. In turn, internalized filial values guide and elevate intergenerational interactions, reinforcing emotional connections. This dynamic cyclical mechanism resonates with both the emphasis on emotional interdependence in Family Systems Theory[45] and the focus on normative integration in Intergenerational Solidarity Theory[60]. It thus highlights that the development of positive age beliefs is the product of the synergistic interplay between affective solidarity and normative solidarity in micro-level family practices.”

Thirdly, Advancing Intergenerational Communication Research

Existing aging research in China has primarily focused on the elderly population (e.g., health, social integration), while overlooking young adults’ perceptions of aging (Zhou & Xie, 2021). By shifting the research focus to young adults, this study identifies dual pathways (daily/ritualistic communication) and a chain mediating mechanism (intergenerational relations→filial piety) that influence their aging imagination, providing a novel theoretical framework for understanding intergenerational interactions within Chinese families.

**【The specific content is on Lines 116 to 127 of the text】**

**“**However, several limitations persist in this field: First, most studies have primarily focused on the elderly population, with less attention paid to how young people acquire knowledge about aging through intergenerational interactions. Second, many studies focus on one-way intergenerational support(e.g., material or emotional support between generations), without exploring the differentiated impact of the forms of interactions (such as daily versus ritualized interactions). Third, the psychological mechanisms underlying intergenerational interactions remain unclear, particularly whether the quality of intergenerational relations(e.g., emotional closeness) and cultural values(e.g., filial piety) play a mediating role, which still requires further examination. In light of these gaps, this study is based on the Chinese family context and investigates the impact of grandparent-grandchild interactions on the younger generation’s perceptions of aging, as well as the mechanisms through which these effects arise.”

Fourthly, Practical Implications for China’s Aging Society

With projections indicating that one-third of China’s population will be elderly by 2050, fostering young adults’ positive attitudes toward aging is critical for social harmony. Our findings offer culturally adaptive interventions aligned with Chinese values (e.g., intergenerational co-learning, transforming family rituals into aging education vehicles), addressing the urgent need for aging education in China. Drawing on cultural and theoretical perspectives, we supplement concrete, actionable recommendations for aging education in Chinese families, schools, and communities, grounded in intergenerational learning (Kakenbosch, Elsa Annette, 2025) and digital feedback (Zhou & Ding, 2020). These include family-based co-learning models, school-based “Grandparent Week” activities, and ritual transformation (e.g., integrating life narrative sessions into Spring Festival gatherings), ensuring the translation of research findings into tangible impact.

**【The specific content is on Lines 768 to 802 of the text】**

“This study further offers implications for aging education in Chinese society. As an emotional communication strategy for intergenerational interactions, intergenerational learning programs have been implemented in numerous countries......Schools can also encourage the organization of “Grandparents’day” events, engaging multiple generations in interactive activities to cultivate mutual respect and empathy across age groups”

**——Next, we address the comments raised by the second reviewer.**

***Reviewer #2: The manuscript examines how grandparent–grandchild daily and ritualistic communication relate to Chinese youths’ “aging imagination,” mediated by intergenerational relations and filial piety. This is timely and relevant to gerontology, family sociology, and communication studies. However, this manuscript need to strengthen construct clarity, measurement validity, analytic rigor (controls, robustness), and reporting transparency. Reframe causal language, fix table inconsistencies, and improve data availability.***

***Rsponse:***

We have enhanced the clarity of the theoretical framework, the validity of the measurements, the rigor of the analyses (including the control variables and robustness tests), and the transparency of the reporting, while also correcting the inconsistencies in the tables. Our dataset was uploaded to the submission system at the time of manuscript submission, data links are now available. Next, we address the reviewers’ comments one by one.

***Comment 1.Conceptual clarity and theoretical framing. Regarding the definition and scope of “aging imagination,” the construct appears to mix self-oriented imagined aging (e.g., planning, acceptance) with broader attitudes toward aging. The current description does not distinguish it from adjacent constructs (e.g., ageism, aging anxiety, future time perspective). I suggest providing a working definition, explicitly contrasting it with related constructs, and including a brief theoretical model figure that positions aging imagination among them.***

***Response:***

Thank you for your valuable and insightful comments. We have carefully addressed your comment, and the detailed revisions are as follows:

1. Supplemented the Definition of "Aging Imagination"

We have clearly defined "aging imagination" based on the Self-Perceptions of Aging (SPA) framework. It is conceptualized as "individuals’ psychological representations and narrative projections of their future elderly states, encompassing anticipated physical changes, social roles, life trajectories, and the meaning of late life". This definition highlights the future-oriented and imaginative characteristics of the construct.

**【The specific content is on Lines 55–77 of the text】**

“In the Chinese context, researchers have proposed the concept of “aging imagination”, yet its specific connotations remain undelineated......Aging imagination serves as a preparatory self-cognitive framework that connects societal dominant perceptions of aging, individuals’ personal views on aging, and behaviors when confronting aging.”

2. Distinguished "Aging Imagination" from Adjacent Constructs

We have systematically compared "aging imagination" with four related constructs (attitudes toward aging, ageism, aging anxiety, and future time perspective) from three dimensions: orientation, temporal focus, and core features. Specifically:

Attitudes toward aging are present-oriented evaluative judgments about aging or the elderly group, which do not involve personal imagination of future old age;

Ageism refers to negative stereotypes and discriminatory behaviors toward older adults as a social category, focusing on group attitudes rather than individual self-perceptions;

Aging anxiety centers on emotional fear and distress toward one’s own aging, lacking structured expectations and preparatory behaviors;

Future time perspective reflects the perceived openness or limitation of the remaining lifespan, without elaborating on the specific connotations of aging-related self-identity and emotional acceptance.

These distinctions have been elaborated in detail in the revised manuscript to clarify the unique theoretical position of "aging imagination".We have included a table (Table 1) summarizing the key differences between "aging imagination" and related constructs, making the distinctions more intuitive and easy to understand. Additionally, we have supplemented a brief theoretical explanation to illustrate the position of "aging imagination" in the existing theoretical system: it extends the SPA framework by focusing on the psychological process of constructing future aging self-images through imagination and prospection, filling the theoretical gap in the formation and integration of aging-related expectations**.**

**【The specific content is on Lines 78–105 of the text】**

“Aging imagination is closely related to several well-established concepts in gerontology, yet it differs from them......Table 1 Comparison of Related Concepts”

All revisions have been seamlessly integrated into the introduction section of the manuscript, maintaining the logical coherence and academic rigor of the original text. We believe these modifications have effectively addressed your concerns about conceptual clarity and theoretical framing, enhancing the overall quality of the study.

***Comment2.The authors argue that daily communication has stronger effects than ritualistic communication, primarily on frequency grounds. They should expand the mechanism by drawing on social learning, intimacy, and role reciprocity; explicitly hypothesize moderators that shape relative effects (e.g., co-residence, geographic proximity, migration status, digital media substitution for rituals, quality of family climate); and add a paragraph explaining how ritualistic contact can be potent in value-activation contexts (e.g., during key rites), potentially boosting the normative (authoritarian) component of filial piety.***

***Response***

1. Expanding Differential Influence Mechanisms

We deepened the explanation of the differential impacts of the two communication forms by integrating Social Learning Theory, Intimate Relationship Theory, and Role Reciprocity Theory.

From the perspective of Social Learning Theory, daily communication provides young adults with high-frequency, authentic observational contexts. The aging coping strategies and positive life attitudes demonstrated by grandparents in daily interactions serve as directly imitable behavioral models for young people. This contextualized learning is direct and sustained, helping young adults gradually internalize the perception that "aging is a natural continuation of life."

Based on Intimate Relationship Theory, high-frequency, informal daily interactions are more likely to strengthen intergenerational emotional trust and psychological bonding. This enables young adults to gain a deeper understanding of elderly life and restructure their cognitive frameworks regarding aging.

From the lens of Role Reciprocity Theory, mutual support in daily communication (e.g., grandparents caring for younger generations and younger generations accompanying grandparents to medical appointments) reinforces intergenerational functional solidarity. It allows young adults to intuitively perceive that aging is not a one-way dependency process but a valuable life stage.

**【The specific content is on Lines 536–563 of the text】**

“The positive impacts of daily interaction on young adults’ aging imagination. Social Learning Theory posits that individuals form cognitions and attitudes through observing and imitating others’ behaviors, as well as receiving environmental feedback......Such interactions have emerged as new modes of intergenerational engagement in everyday life and serve as a vital supplement to family communication”

2. Clarifying Potential Moderating Variables

Drawing on literature related to family contexts and structural characteristics, we proposed and discussed potential moderating variables. Since these variables were not measured in this study, we explicitly note that this constitutes an area for future research:

Residential arrangement and geographic proximity: Co-residence or close-distance living enhances the accessibility and emotional intensity of daily communication, while long-distance separation may assign ritualistic communication a stronger emotional compensation function.

Migration status and family mobility: For young migrant groups, digital media serves as a crucial supplement to face-to-face communication, fostering new forms of digital rituals that in turn influence communication outcomes.

Regarding your third suggestion that "Family atmosphere and the quality of intergenerational relations: interactions in harmonious families are more likely to produce positive learning effects, while interactions in families with strained relations may merely serve as a tool to maintain superficial bonds", we highly appreciate this valuable comment. We acknowledge that this is indeed a potential influencing factor; however, intergenerational relations have already been analyzed as a mediating variable in this study, and thus we have not further examined it as a potential moderating variable.

**【The specific content is on Lines 586–596 of the text】**

“Notably, the relative effects of these two communication forms may be moderated by a range of family contextual and structural variables, an area warranting further in-depth exploration in future research. First, residential arrangements and geographic proximity: Co-residence or living in close proximity is likely to significantly enhance the accessibility and emotional intensity of daily interactions[63], thereby influencing interactions effects. In contrast, long-distance separation may compel ritualistic interactions (e.g., returning to one’s hometown during the Spring Festival) to fulfill a stronger emotional compensation function [64].Second, migration status and family mobility: For young migrants or family members living away from their hometowns, digital media serves as a crucial supplement to face-to-face communication, fostering the emergence of new digital rituals and thereby shaping interactions outcomes”.

3. Supplementing the Value Activation Role of Ritualistic Communication

We added a new paragraph to elaborate on the unique value of ritualistic communication in specific contexts: During ritual occasions such as the Spring Festival, a series of structured symbolic behaviors transform abstract filial piety ethics into concrete practices. This effectively activates and reinforces the component of filial piety beliefs—namely, adherence to grandparents’ authority and fulfillment of ritual obligations.

**【The specific content is on Lines 564–585 of the text】**

“Ritual interaction also exerts an undeniable shaping effect on young adults’ aging imagination. In Chinese contexts, rituals such as the elderly’s birthdays and traditional festivals often extend beyond the nuclear family, evolving into collective events that incorporate all participating relatives into the broader “family” category[58]......Long-term engagement and observation allow young adults to witness the ongoing contributions of elders within the family, which in turn activates their concept of filial piety and helps them recognize that old age is not merely a stage of one-way dependence and demand, but a period still endowed with the potential to create emotional and cultural value [62].”

Simultaneously, we objectively analyzed potential risks: Overemphasizing grandparents’ authoritarian status and ritualized interactions may impose moral pressure on young adults, which in turn triggers negative perceptions of aging. This makes the research discussion more comprehensive.This part is elaborated in the section discussing the mediating role of the concept of filial piety.To avoid redundancy, we present this content only once

**【The specific content is on Lines 658–662 of the text】**

“At the same time, some scholars argue that filial piety in China exerts a certain degree of pressure on young people. Overemphasizing the elderly’s authoritative status and ritualized intergenerational communication may also impose moral burdens on young individuals, under such pressure,, the positive impact of ritual interactions is undermined[71]. ”

***Comment3.The authors discuss filial piety as a dual construct (reciprocal vs. authoritarian) but analyze it as a single composite. They should separate and report the two subdimensions.***

***Response:***

Thank you very much for this insightful comment. We appreciate the reviewer’s careful attention to the conceptualization and operationalization of filial piety.

We agree that the Dual Filial Piety Model (DFPM) theoretically distinguishes between reciprocal filial piety (RFP) and authoritarian filial piety (AFP) as two motivational sources underlying filial behaviors (Yeh & Bedford, 2003). This theoretical distinction has been highly influential and is particularly important for studies aiming to compare the differential psychological outcomes associated with these two motivations.

However, in the present study, we intentionally treated filial piety as a composite construct, rather than disaggregating it into two subdimensions, for both theoretical and analytical reasons.

First, although Yeh and Bedford distinguish filial piety as dual at the motivational level, they do not conceptualize RFP and AFP as mutually exclusive or oppositional constructs. Rather, their subsequent work explicitly emphasizes that the two motivations can co-exist within individuals and are jointly embedded in everyday filial practices, reflecting the culturally integrated nature of filial piety in Chinese contexts (Bedford & Yeh, 2019). From this perspective, the “dual” nature of filial piety refers to analytically distinguishable motivational schemas rather than empirically separable or competing value systems.

Second, the primary aim of our study is not to contrast the distinct effects of reciprocal versus authoritarian filial piety, but to examine how overall filial piety as a culturally embedded value orientation is shaped through intergenerational communication and how it relates to young adults’ aging imagination. When the research focus concerns the general endorsement of filial ideology, several scholars have argued that treating filial piety as an integrated construct more accurately reflects its holistic role in Chinese family life (Ho, 1996; Bedford & Yeh, 2019).Under such conditions, employing a composite construct can be theoretically defensible and methodologically parsimonious, particularly when the research goal is not to disentangle distinct motivational pathways.

To address the reviewer’s concern and to avoid potential ambiguity, we have revised the manuscript to explicitly clarify our theoretical position regarding the composite treatment of filial piety. Specifically, we have added a paragraph in the Discussion section explaining that, although we draw on the DFPM framework for conceptual grounding, our analytical choice reflects an intentional focus on the integrated and co-existing nature of filial piety in Chinese cultural contexts. Relevant authoritative literature has been added accordingly

**【The specific content is on Lines 357–364 of the text，Purple font】**

“Yeh distinguishes Confucian filial piety into reciprocal filial piety and authoritarian filial piety, but he does not conceptualize these two constructs as opposing or mutually exclusive value systems [46].In their subsequent research, the authors explicitly emphasized that these two filial piety motivations often coexist within the same individual and are co-embedded and intertwined in daily family practices, thus reflecting the integrative nature of filial piety in the Chinese cultural context [47]. When researching filial piety beliefs, scholars have noted that treating filial piety as an integrative construct better reflects its holistic cultural functions in Chinese family life[47](Ho, 1996; Bedford & Yeh, 2019).

We hope this clarification addresses the reviewer’s concern and demonstrates that the use of a composite filial piety construct in this study is a theoretically informed and purposeful choice rather than an oversight.

***Comment4 The manuscript does not include full item wordings for all constructs, and the reported psychometric evidence is limited. Please provide full item lists and additional validity evidence.***

***Response：***

Regarding the reviewer's suggestion to list complete items, we have already described the specific items of each scale in various sections of Measures. **For specific items, refer to the red text in the Measures section**

Daily interactions**:**“The scale measuring daily interactions between grandparents and grandchildren：Respondents were asked to answer four items on a 5-point Likert scale, which included: chatting with (paternal/maternal) grandparents, going shopping with (paternal/maternal) grandparents, accompanying (paternal/maternal) grandparents to the doctor, and having meals with (paternal/maternal) grandparents”**(Lines 334–338)**

ritual interactions**:**“Respondents were required to answer two items on a 5-point Likert scale, specifically: having celebrated the birthday of (paternal/maternal) grandparents, and celebrating traditional family reunion festivals such as Chinese New Year with (paternal/maternal) grandparents.”**(lines 343-345)**

intergenerational relations**：**“The scale for measuring intergenerational relations was adapted from the version developed by Wang Honglei et al(Wang, Tan, & Bao, 2023). Respondents answered three items on a 5-point Likert scale, including: emotional closeness to (paternal/maternal) grandparents, having a good relationship with(paternal/maternal) grandparents, and willingness to listen when(paternal/maternal) grandparents have concerns or difficulties.”**(Lines 349-351)**

Filial Piety:“paying attention to the health of the elderly, engaging in conversations with the elderly to understand their thoughts and feelings, being mindful of the daily life of the elderly, showing care and concern for the elderly, providing for the elderly to improve their comfort, feeling gratitude for the care received from the elderly, attending the funeral of the elderly regardless of the distance, helping the elderly when they are busy, yielding to the elderly when there is disagreement, handing over all earnings to the elderly before marriage, breaking promises to friends to comply with the elderly, giving up personal ambitions to fulfill the wishes of the elderly, promptly carrying out any task assigned by the elderly, avoiding marriage to someone disapproved of by the elderly, ensuring the continuation of the family line by having at least one son, and living with the elderly after marriage.**”(lines 368-377)**

Self-Imagined Aging Scale:“ being mentally prepared for the possibility of living a single life in old age, accepting the aging process and the changes in appearance, maintaining an optimistic view of aging, planning various strategies for the expected life in old age, and accumulating resources to achieve personal goals for aging”**(lines 384-387)**

The reliability of all scales was reported in the initial draft, and we now supplement data/analyses related to the scales’ validity. Among them,the KMO value of the scale "Daily interactions between Grandparents and Grandchildren" is 0.778. Bartlett’s Test of Sphericity was significant, with an approximate chi-square value of 1919.863 (p < .001).The KMO value of the scale "Intergenerational relations between Grandparents and Grandchildren"is 0.759. Bartlett’s Test of Sphericity was significant, with an approximate chi-square value of 1750.095 (p < .001).The KMO value of the scale Concept of "Filial Piety"is 0.935. Bartlett’s Test of Sphericity was significant, with an approximate chi-square value of 11890.020 (p < .001).The KMO value of the scale "Self-Imagined Aging Scale"is 0.758. Bartlett’s Test of Sphericity was significant, with an approximate chi-square value of 1819.217 (p < .001).

**For specific content, refer to the blue text in “Measures”.**

***Comment5. Harman’s single-factor test is inadequate on its own. Add a CFA-based test comparing a one-factor model with the proposed multi-factor model and report the change in fit indices.***

***Response：***

In response to the reviewer's suggestions for revision, we have added a test based on CFA on top of Harman's one-factor test.In response to the reviewer's suggestions for revision, we have added a test based on CFA on top of Harman's one-factor test.The results indicated a poor fit to the data, with a chi-square to degrees of freedom ratio (χ²/df) of 30.557, exceeding the recommended cutoff of 5. In addition, the goodness-of-fit index (GFI = 0.347), comparative fit index (CFI = 0.375), and Tucker–Lewis index (TLI = 0.329) were all below the acceptable threshold of 0.90, while the root mean square error of approximation (RMSEA = 0.195) exceeded the recommended upper limit of 0.08. Collectively, these findings demonstrate that the measurement items could not be adequately represented by a single factor, indicating that common method bias was not a serious concern in the present study.

**【The content can be found in lines 395-408】**

“In this study, Harman’s single-factor test was employed to examine common method bias. Results indicated that all five factor eigenvalues exceeded 1, with the first factor accounting for only 29.31% of total variance, which is below the critical threshold of 40%. Secondly, given that the single-factor test may yield non-significant results under certain conditions, all measurement items corresponding to the study variables were loaded onto a single latent factor. A single-factor model was subsequently estimated using AMOS 26.0 to examine model fit. The results indicated a poor fit to the data, with a chi-square to degrees of freedom ratio (χ²/df) of 30.557, exceeding the recommended cutoff of 5. In addition, the goodness-of-fit index (GFI = 0.347), comparative fit index (CFI = 0.375), and Tucker–Lewis index (TLI = 0.329) were all below the acceptable threshold of 0.90, while the root mean square error of approximation (RMSEA = 0.195) exceeded the recommended upper limit of 0.08. Collectively, these findings demonstrate that the measurement items could not be adequately represented by a single factor, indicating that common method bias was not a serious concern in the present study.”

***Comment6. No covariates are included, so potential confounds remain. Report models with and without controls to demonstrate robustness of the mediation effects. At a minimum, include plausible confounders and family-structure controls. Research design and inference should reflect these additions.***

***Response：***

In response to the reviewer's comment that the study did not incorporate covariates, we added six control variables related to gender, age, education, home location, occupation, and communication duration to the research model. The results showed that compared to the model without any control variables, the direction, magnitude, and significance of the key path coefficients were highly consistent, indicating that the chain mediation mechanism discovered in this study is robust. The table in the article lists the path coefficients of the model with control variables, and the table of path coefficients for the model without any control variables is provided as supplementary material at the end of the article.It should be noted that the reviewers suggested incorporating family structure as a control variable. However, this factor was not considered during the questionnaire design phase, so we are unable to include it as a control variable. Nevertheless, we measured a similar variable—communication duration with grandparents—and incorporated it into the data analysis as a control variable, which we believe also supports the validation of our research questions. We acknowledge the oversight and inadequate consideration of family structure, and thus we have acknowledged this limitation in the "Study Limitations" section. We will further examine the impacts of such factors in future investigations.

**【The textual descriptions in this paper are presented in Lines 459–464 of the text】**

“To provide a more conservative estimate of the mediation effects, all results reported in the main text are based on models controlling for gender, age, education,family location, occupation, ,and communication duration with grandparents. For robustness checks, we also estimated the same serial mediation model without covariates, and the results were substantively similar. The corresponding results are reported in S1 Table and S2 Table in the Supporting information.”

【**Lines 831–834 of the text**】

“Meanwhile, this study did not measure variables such as family structure, residential distance, and digital media communication. Future research should fully consider the impacts of these factors on the model to gain a more comprehensive understanding of the relationship between grandparent-grandchild family communication and young adults’ aging imagination”.

***Comment7. There is an inconsistency about recruiting “experts and practitioners” versus “young people,” and the exclusion criteria are vague. Please clarify the sampling frame and operationalize exclusions.***

***Response：***

In response to the reviewer's comments, we carefully reviewed the content of the article and found errors in the recruitment of "experts and practitioners" and "young people" written in the article, which have now been corrected.This is a spelling error. "Experts and practitioners" has been corrected to participants. Regarding the exclusion criteria in our study, if the questionnaire response is incomplete and exhibits regularity (i.e., the answers are the same or have a specific pattern) and the response time is less than 2 minutes, it indicates that the questionnaire is invalid.

**【The specific content is on lines 318-329 of the text】.**

“The survey began to recruit participants on March 1, 2024 and ended on July 31...... Among the participants, 256 were male (32.8%) and 522 were female (67.2%). In terms of age distribution, 476 participants were aged 18-23, accounting for 61.18% of the sample, 191 were aged 24-29, representing 24.55%, and 111 were aged 30-35, comprising 14.27%”

***Comment8. In Tables 5 and 6, the direct-effect confidence intervals are inconsistent with the point estimates, and it is unclear whether coefficients are standardized or unstandardized. Please correct and clarify.***

***Response：***

Due to the addition of one table during revision, Tables 5 and 6 in the text have been updated to Tables 6 and 7. The data in both tables have been corrected, and the coefficients have been standardized.The data were cross-verified by two staff members to ensure accuracy.The specific details are provided in Tables 6 and 7 in the document, which we have also included here for your reference.

**Table 6 The chain mediation of interpersonal relations and filial piety among grandchildren and youths(Model 1)**

|  |  |  |  | **95%CI** | |
| --- | --- | --- | --- | --- | --- |
| **Model** | **Intermediary paths for variables** | **Standardized effect size** | **BootSE** | **BootLLCI** | **BootULCI** |
| Model 1 | D.G.G.I→Y.P.I.A(Total Effect) | 0.2749 | 0.0366 | 0.2031 | 0.3467 |
|  | D.G.G.I→Y.P.I.A(Total Direct effect) | 0.1507 | 0.0410 | 0.0702 | 0.2312 |
|  | Total Indirect Effect | 0.1242 | 0.0283 | 0.0700 | 0.1827 |
|  | D.G.G.I →I.R →Y.P.I.A | 0.0762 | 0.0234 | 0.0306 | 0.1236 |
|  | D.G.G.I→F.P→Y.P.I.A | 0.0325 | 0.0123 | 0.0120 | 0.0612 |
|  | D.G.G.I→I.R→F.P→Y.P.I.A | 0.0155 | 0.0061 | 0.0060 | 0.0295 |

Note. D.G.G.I,Daily grandparent-grandchild interactions; Y.P.I.A,Young people’s imagination of aging; I.R,Intergenerational relations; F.P,filial piety

**Table 7 The chain mediation of interpersonal relations and filial piety among grandchildren and youths（Model 2）**

|  |  |  |  | **95%CI** | |
| --- | --- | --- | --- | --- | --- |
| **Model** | **Intermediary paths for variables** | **Standardized effect size** | **BootSE** | **BootLLCI** | **BootULCI** |
| Model 2 | R.G.G.I→Y.P.I.A（Total Effect） | 0.2379 | 0.0362 | 0.1668 | 0.3090 |
|  | R.G.G.I→Y.P.I.A(Total Direct effect) | 0.1380 | 0.0382 | 0.0629 | 0.2131 |
|  | Total Indirect Effect | 0.0999 | 0.0225 | 0.0593 | 0.1470 |
|  | R.G.G.I→I.R→Y.P.I.A | 0.0674 | 0.0192 | 0.0324 | 0.1079 |
|  | R.G.G.I→F.P→Y.P.I.A | 0.0143 | 0.0083 | 0.0005 | 0.0328 |
|  | R.G.G.I→I.R→F.P→Y.P.I.A | 0.0182 | 0.0062 | 0.0080 | 0.0319 |

Note. R.G.G.I,Ritualistic grandparent-grandchild interactions;Y.P.I.A,Young people's imagination of aging;I.R,Intergenerational relations; F.P,filial piety

***Comment9.Avoid over-interpretation and offer more nuanced discussion. Recast the discussion to emphasize associations and plausible mechanisms. Integrate literature on digital intergenerational contact (e.g., whether WeChat-based contact substitutes for or complements face-to-face interactions), and note potential negative dynamics (e.g., ritual obligations perceived as pressure may undermine positive aging imagination for some youth).***

***Response：***

We have carefully revised the manuscript accordingly. Below is a detailed account of the modifications and responses to your feedback:

1. Avoid over-interpretation and strengthen the refinement of discussion revisions

We have nearly rewritten the entire Discussion section. We recognize that the initial draft contained overly hasty causal inferences, a flaw that has been thoroughly addressed in the revised version. The core focus of this section has shifted from explaining why ritualistic and daily interactions produce different outcomes to systematically elaborating on the associations between these two types of interactions and young adults’ attitudes toward aging.

In the section on intergenerational relations, we argue that present study reveals the psychological pathway through which intergenerational interactions influence young people’s aging imagination: communication behaviors first affect the quality of emotional bonds between generations (i.e., intergenerational relationships), which in turn shapes young adults’ future perceptions of their own aging via these emotional ties. This discovery advances research beyond a sole focus on interaction frequency or forms to an exploration of the relational dynamics embedded in such interactions. Furthermore, the prevalent phenomenon of “grandparent-grandchild bond” in Chinese society provides a unique and crucial cultural context for understanding this psychological mechanism. Regarding the mediating role of filial piety, we have emphasized both the activating effects and potential negative dynamics of ritualistic interactions in the context of Chinese ritual culture. All arguments in this section are strictly grounded in existing academic literature and theoretical frameworks, replacing the previous imprecise causal claims with evidence-based reasoning.

We have also adjusted the overall linguistic style and analytical approach of the Discussion section to ensure greater conceptual rigor and academic prudence.

2. We have systematically incorporated relevant research on digital intergenerational interactions into Section "Dual pathways of grandparent-grandchild interaction shaping young adults’ aging imagination: daily interaction vs ritualistic interaction" .

We added discussions on how information and communication technologies (ICTs) such as social media and smart devices reshape family interactions in Chinese contexts (Chen, 2022). Studies confirm that mediated interactions (e.g., WeChat family groups, video calls) have become important supplements to face-to-face communication, especially for geographically separated families, maintaining frequent intergenerational connections and compensating for the lack of offline ritualistic interactions (Su et al., 2024; An & Wang, 2020; Wu & Long, 2016).

**【See lines 555–563 in the text】**

“With the penetration of digital media into Chinese families, information and communication technologies (ICTs), such as social media and smart monitoring systems, are reshaping family interactions and daily life by constructing increasingly sophisticated social scenarios, a process infused with the interplay of emotions and power [54]. From urban to rural contexts, and from quantitative panel data analyses to qualitative in-depth interviews, studies have repeatedly confirmed that mediated interactions with Chinese characteristics exert a significant impact on intergenerational relationships. Such interactions have emerged as new modes of intergenerational engagement in everyday life and serve as a vital supplement to family communication[55] [56] [57].”

3.Supplementation of Discussions on Potential Negative Dynamics

We expanded the analysis of potential negative impacts of ritualistic interactions in Section 4.1,

We primarily supplemented the following literature for discussion：Moral pressure from ritual obligations: Overly formalized ritualistic interactions (e.g., mandatory participation in family gatherings, rigid performance of filial piety rituals) may be perceived as moral pressure by young adults (Yin & Wu, 2019). This pressure can trigger psychological resistance, leading young people to perceive elders as rigid and authoritarian, and generalizing such perceptions into negative stereotypes of aging (e.g., "aging leads to stubbornness and poor communication").

Since the negative effects of ritual interaction are not the core issue of this study, we consider them as a supplementary discussion.

**【See lines 658–662 in the text】**

“At the same time, some scholars argue that filial piety in China exerts a certain degree of pressure on young people. Overemphasizing the elderly’s authoritative status and ritualized intergenerational communication may also impose moral burdens on young individuals, under such pressure, the positive impact of ritual interactions is undermined[71].”

All revised content is supported by relevant literature citations, ensuring the academic rigor of the arguments. We believe these modifications have effectively addressed your concerns, making the discussion more balanced, nuanced.

***Comment10*. *Provide practical recommendations with guardrails. For example, in educational interventions, propose short, evidence-based modules that promote reflective dialogue with grandparents (e.g., guided oral history, co-learning technology tasks), rather than merely advocating “more contact.”***

***Response:***

We have addressed the final paragraph of Section “Theoretical Contributions and Practical Implications”, where specific measures are proposed based on the relevant literature on intergenerational learning programs and the findings of this study. These measures, grounded in theoretical frameworks and tailored to China’s contextual realities. As an "emotional communication strategy" for intergenerational interaction, intergenerational learning programs have been implemented in numerous countries (Newman & Hatton-Yeo, 2008). Intergenerational learning refers to a reciprocal and integrative learning model that engages individuals of different age groups. By encouraging cross-age collaboration and learning, it facilitates the exchange of ideas, emotions, experiences, and professional skills across generations, thereby enabling the acquisition of skills, knowledge, and values.

Currently, intergenerational learning is also emerging quietly in China. In their research on digital support (i.e., younger generations assisting older ones with digital technologies), Chinese scholars have found that intergenerational learning centered on digital support within Chinese families constitutes a two-way interactive process. The guidance provided by younger generations to their elders reflects mutual influence among two or even three generations, shaped by emotional bonds, intergenerational relationships, and power dynamics. Digital support fosters "processual intimacy" between generations, which further enhances mutual understanding, emotional alignment, and ideological consensus across age groups (Zhou & Ding, 2020). Research conducted in Taiwan has similarly indicated that the life narratives of older adults can effectively narrow the emotional distance between generations (Zhou & Xie, 2021).

Despite its incipient development in the Chinese context, intergenerational learning has not yet been widely promoted. Practical steps may include designing intergenerational learning programs themed around digital support and oral life history, and integrating such programs into school education to create more natural, equitable, and harmonious opportunities for interaction between older and younger generations. Families, on a voluntary and equal basis, can promote the "grandparent-grandchild co-learning" model, encouraging young people to assist their elders in mastering smart devices. Meanwhile, traditional family rituals, such as Spring Festival and birthday celebrations, can be transformed into carriers of aging education by incorporating sessions for sharing life narratives, allowing older family members to recount their life stories. Schools, for their part, should incorporate aging education into the national education system, offering life education courses. Through activities like picture book reading and role-playing, students can develop an understanding of the wholeness of the life course. Additionally, schools may encourage the organization of "Grandparents’ Week" events, where individuals of different generations participate together to cultivate mutual respect and empathy across age groups.

**【See lines 768–802 in the text】**

“ This study further offers implications for aging education in Chinese society. As an emotional communication strategy for intergenerational interactions, intergenerational learning programs have been implemented in numerous countries......Schools can also encourage the organization of “Grandparents’day” events, engaging multiple generations in interactive activities to cultivate mutual respect and empathy across age groups.*”*

**Comment11.Add the IRB approval number/code if available.**

***Response：***

We have added the Institutional Review Board (IRB) approval number, which is 20240002.

***Comment12.Regarding writing and formatting, clean up spacing and punctuation throughout (e.g., change “relations ,” and “youths’ constructive” to “relations,” and “youths’ constructive”).***

***Response:***

Thank you sincerely for pointing out the issues regarding writing format and punctuation in the manuscript. Regarding the spacing and punctuation issues you mentioned, we have carefully reviewed and cleaned up the entire manuscript page by page and sentence by sentence:

Uniformly corrected spacing errors around punctuation marks. For example, expressions such as "relations ," and "youths’ constructive" have been standardized to "relations," and "youths’constructive," ensuring that the use of commas, apostrophes, and other punctuation marks complies with academic writing format requirements.

Conducted a thorough check of paragraph spacing, font formatting, citation annotations, and other details throughout the manuscript, corrected any potential inconsistent formatting issues, and ensured the manuscript is neat and standardized in format.

Focused on verifying the consistency of punctuation usage in various sections including the abstract, keywords, main text, and references, to avoid similar formatting omissions.

After systematic revision, the manuscript has been significantly improved in terms of writing format and punctuation standardization. We attach great importance to the rigor of academic writing.

We believe that these extensive revisions have significantly improved the clarity, rigor, and contribution of the manuscript, and we hope that the revised version now meets the standards for publication in PLOS ONE. We are grateful for the constructive feedback, which has greatly strengthened our work.If there are any further revisions you deem necessary, we welcome you to contact us anytime, and we will be glad to address them promptly.

Thank you again for your consideration.

Sincerely,

Fen Xie

On behalf of all authors
